# What Differs on the Enzymatic Acetylation Mechanisms for Arylamines and Arylhydrazines Substrates? A Theoretical Study

**DOI:** 10.1155/2009/783035

**Published:** 2009-08-06

**Authors:** Qing-An Qiao, Xiao-Min Sun, Jie Jing, Xin Chen, Hua-Yang Wang, Chuan-Lu Yang, Zheng-Ting Cai

**Affiliations:** ^1^School of Chemistry and Materials Science, Ludong University, Yantai 264025, China; ^2^Environment Research Institute, Shandong University, Jinan 250100, China; ^3^School of Physics and Electronic Engineering, Ludong University, Yantai 264025, China; ^4^Institute of Theoretical Chemistry, Shandong University, Jinan, Shandong 250100, China

## Abstract

The acetylation mechanisms of several selected typical substrates from experiments, including arylamines and arylhydrazines, are investigated with the density functional theory in this paper. The results indicate that all the transition states are characterized by a four-membered ring structure, and hydralazine (HDZ) is the most potent substrate. The bioactivity for all the compounds is increased in a sequence of PABA ≈ 4-AS < 4-MA < 5-AS ≈ INH < HDZ. The conjunction effect and the delocalization of the lone pairs of N atom play a key role in the reaction. All the results are consistent with the experimental data.

## 1. Introduction

**Scheme 1:**
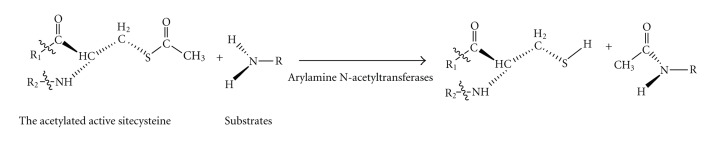
The NATs catalyzed acetyl transfer reaction.

The arylamine N-acetyltransferases (NATs, EC 2.3.1.5) are phase II metabolism enzymes found in both prokaryotes and eukaryotes [1]. The N-acetylation reaction leads to the detoxification of arylamine xenobiotics and finally directs to electrophilic arylnitrenium ions, which are considered to be responsible for DNA adduct formation [2, 3]. To Humans, the two functional NAT isozymes, NAT1 and NAT2, show great differences in substrate specificity and tissue distribution in spite of 81% amino acid sequence identity [4]. The latter, NAT2, is expressed predominantly in liver and intestinal epithelium [5]. Previous studies [2, 6, 7] have supposed that NATs catalyze an acetyl transfer by a classical ping-pong kinetic mechanism (Scheme 1). Site-directed mutagenesis analysis of human NAT2 and *Salmonella typhimurium* NAT (StNAT) [7, 8] suggested that a cysteine residue in the active site was responsible for mediating the acetylation process. A recent presteady-state and steady-state kinetic studies on *p*-nitrophenyl acetate (PNPA) and NAT2 [9, 10] revealed that the catalytic mechanism of NAT2 might depend on the formation of a thiolate-imidazolium pair. Though the enzyme is found in both eukaryotes and prokaryotes, the endogenous roles of NATs are still unclear [11]. The substrate determination revealed that both arylamines and arylhydrazines could be acetylated by NATs [11–15]. The required steps in the acetyl transfer reaction are composed of the acetyl group transfer from the active site cysteine residue to the substrate, and the removal of one proton from the latter to the former. In this paper, a detailed theoretical study on the behaviors of arylamines and arylhydrazines substrates in acetylation is available, including the properties of their structures, the transition states, the profiles of energies.

## 2. Methodologies

All calculations were performed with the density functional theory (DFT) B3LYP [16–18] method as implemented in Gaussian03 program package [19], which has previously been successfully employed on a number of enzymatic systems [20]. When 6-31G* and 6-311+G (3df, 2p) basis sets were used, the B3LYP hybrid functional was preferred to Hartree-Fock (HF) and MP2 methods [21, 22]. Though it sometimes fails in treatment of dispersion-rich interactions, the B3LYP method has been successfully applied on many biological systems [23–25]. 

 The geometries of all the reactants, intermediates, and products are optimized at the B3LYP/6-31G* level of theory. The most stable conformations as well as their energies at every equilibration and transition states have been figured out. Frequency calculations are performed to all the resulting stationary points and each transition states has only one imaginary frequency. Furthermore, MP2/6-311+G** method is employed on the optimized structures of stationary points to get more accuracy energy profiles. If not specially pointed out, all the following energy analyses refer to the results from MP2/6-311+G**//B3LYP/6-31G (d) calculations.

## 3. Results and Discussion

### 3.1. The Substrates' Frontier Obitals

Six substrates, *p-*aminobenzoic acid(PABA), 4-methoxylaniline(4-MA), 4-aminosalicylate (4-AS), 5-aminosalicylate(5-AS), isoniazid(INZ), hydralazine (HDZ), are selected according the references [11–15], which can be divided into two distinct families: arylamines and arylhydrazines. The energies for frontier obitals (including HOMO-2, HOMO-1, HOMO, LUMO, LUMO+1 and LUMO+2) of all six substrates are listed in Table 1, which are considered playing important roles in bioenzymatic systems [26]. For the arylamines, the HOMO energies are reduced in a sequence of 4-MA > 5-AS > PABA > 4-AS, which indicates the increasing of their nucleophilic reactivity. Among all the six substrates (see Figure  1 in the supplementary materials available online at doi: 10.1155/2009/783035), INZ has the largest energy gap between HOMO and LUMO, which suggests its stability. The Natural Population Analysis (NPA) results showed that the active amino N atoms for the arylamines family are more electronegative than those of the hydrazines group, which is mainly caused by the conjugation effect. 

Though PABA and 4-AS have different substitutions on the *p*- site of the amino group at the six-membered ring, their bioactivities are nearly the same. An intermolecular H-bond will stabilize the substrate itself with the energy drop about 19–21 kJ/mol. Both the HOMO and LOMO energies for 5-AS are higher than those of 4-A*S*′s, suggesting that the former is more reactive than the latter. 

 For the arylhydrazines substrates, the lone pair of the N atoms at the backbone of the six-membered ring will be delocalized at the whole system so as to enhance its stability. The *E*_(*L*-*H*)_  values of HDZ is 0.0318 a.u. higher than that of INZ's, indicating its higher reactivity than the latter.

### 3.2. The Different Pathways and Transition States

**Figure 1:**
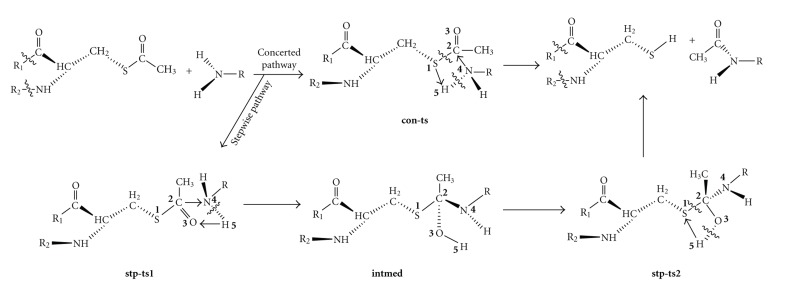
The concerted and stepwise pathways for arylamine N-acetyltransferases catalyzed reaction.

**Figure 2:**
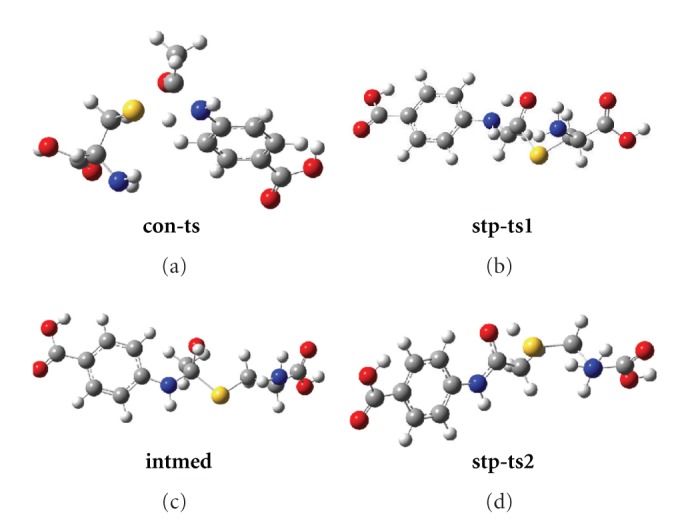
The structure of transition states for both concerted and stepwise pathway of PABA.

In principle, all the substrates can react with the active site cysteine residue via a concerted pathway or a stepwise one. To the former, the transition states (see Figures 1 and 2, **con-ts**) experience a concerted transfer of the hydrogen H5 to S1 atom of cysteine and a bond formation between N4 and C2 atoms. The broken of old bonds (N4H5 and S1C2) and the formation of new ones (C2N4 and S1H5) take place simultaneously. The reactants and the target products are connected by the only transition state on the potential energy surface (PES). The main structure data of all transition states are listed in Table  1 of the supplementary materials. For the stepwise mechanism, H5 atom will be firstly transferred to the O3 atom of the carbonyl group, tending to generate a new bond between N4 and C2 atom via transition state **stw-ts1**. Then, a thiolester intermediate (**intmed**) will be formed. Consequently, the second migration of H5 will take place from the hydroxyl group to S1 atom via **stw-ts2** together with the rupture of S1-C2 bond, which will finally direct to the products. 

The results indicate that all the transition states are characterized by a four-membered ring structure which is nearly planar. There are two small angles less than 80° in every transition state (C2S1H5 and S1C2N4 for ** con-ts**, C2O3H5 and C2N4H5 for **stp-ts1**, C2S1H5 and S1C2O3 for **stp-ts2**), which bring great strain to the whole system and make it unstable. Among the six concerted transition states (**con-ts**) of all the substrates, the properties of the bond C2N4, N4H5 and S1H5 are roughly the same for all the substrates, while the interaction between S1 and C2 is one of the determining factors for the concerted step. 

 Things become different for the stepwise pathways. The hybridization changes of C2 atom follow a similar tendency (*sp* ^3^ → *sp* ^2^ → *sp* ^3^) for all the substrates during the stepwise acetylating (Table 1 supplementary). The first migration of H5 will lead to transition state **stp-ts1**, and then an intermediate named **intmed** is located on the potential energy surface (PES), which is a local minimum. It is a tetrahedral thiolester intermediate as proposed in previous experimental studies [10]. It is short-lived and a consequent H5 transfer will soon occur via transition state **stp-ts2**. For the stepwise pathway, the structures of the traction states for different substrates differ very little with others. The 3D structures of the transition states were listed for PABA (Figure 2), the other ones were similar to those.

### 3.3. The Energies

**Figure 3:**
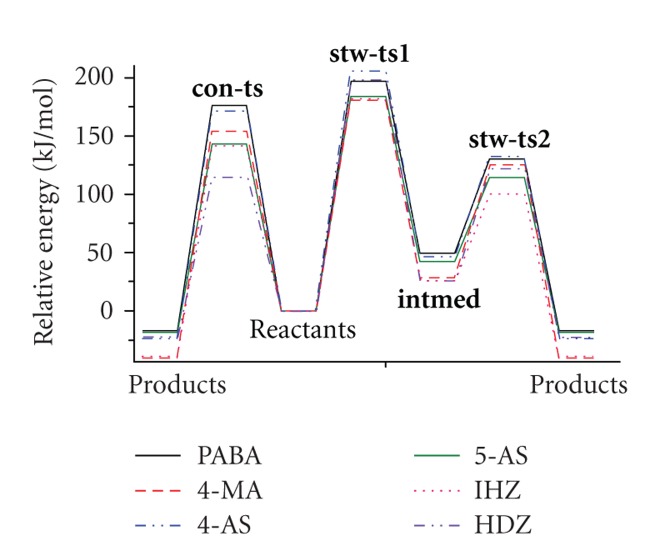
The energy profiles for all substrates.

The relative energies of all possible pathways for the six substrates are carried out based on the energy sum of the reactants taken as zero (Figure 3). From Figure 3, we find that the concerted pathways are favored to the stepwise ones. The energy barriers of concerted transition states **(con-ts)** are lower than those of the stepwise ones **(stp-ts1)** at a range of 83.5 kJ/mol to 26.9 kJ/mol (Table  2 supplementary). The arylhydrazines are better substrates than the arylamines, and HDZ is the most reactive one with the lowest activation energy, which meets the experimental data with good agreement [12]. This conclusion could be also drawn from the structure data analysis (Table 1 supplementary). The enhanced conjunction effect and the delocalization of the nitrogen lone pairs at the backbone stabilized the transition state. The bioactivity for all the substrates is increased in a sequence of PABA ≈ 4 − AS < 4 − MA < 5 − AS ≈ INH < HDZ.

## 4. Conclusions

The following conclusions can be drawn.

- All the substrate can be acetylated via two different pathways: the concerted and the stepwise, and the former is much preferred owing to the lower activation energies.
- From our calculation, arylhydrazines are better substrates than the arylamines, and HDZ is the most reactive one with the lowest activation energy. The bioactivity for all the substrates is increased in a sequence as PABA ≈ 4 − AS < 4 − MA < 5 − AS ≈ INH < HDZ, which is consistent with the experimental results very well [12].
- The conjunction effect and the delocalized lone pairs play very important roles in acetylation. The enhanced conjunction effect and the increasing numbers of the lone pairs at the six-membered ring will lead to the lower energy barrier.

## Supplementary Material

For the length limitation of this paper, the 3D structures of all the arylamines, and arylhydrazines substrates, the main structure data for all the transition states and the relative energies for different pathways were gathered in the supplementary materials.

## Figures and Tables

**Table 1:** The energies of the frontier orbitals.

Energy/a.u.	HOMO-2	HOMO-1	HOMO	LUMO	LUMO+1	LUMO+2	*E*_(*L*-*H*)_^(a)^
PABA	−0.4469	−0.3636	−0.3183	0.0536	0.0660	0.0749	0.3791
4-MA	−0.4366	−0.3398	−0.2890	0.0693	0.0815	0.0863	0.3583
4-AS	−0.4597	−0.3443	−0.3244	0.0525	0.0627	0.0729	0.3769
5-AS	−0.4419	−0.3641	−0.2924	0.0611	0.0619	0.0697	0.3535
INZ	−0.4162	−0.3850	−0.3484	0.0599	0.0655	0.0787	0.4083
HDZ	−0.3822	−0.3664	−0.3171	0.0590	0.0605	0.0656	0.3761

^(**a**)^ *E*_(*L*-*H*)_ refers to the energy difference between HOMO and LUMO.
