# Supplementary material for: What Differs on the Enzymatic Acetylation Mechanisms for Arylamines and Arylhydrazines Substrates? A Theoretical Study
Source: Res Lett Biochem. 2009 Aug 6;2009:783035. doi: 10.1155/2009/783035 (PMC3005951; doi:10.1155/2009/783035)
Supplement: Supplementary file 1 — For the length limitation of this paper, the 3D structures of all the arylamines, and arylhydrazines substrates, the main structure data for all the transition states and the relative energies for different pathways were gathered in the supplementary materials. [file 783035.f1.doc]

**The supplementary materials**


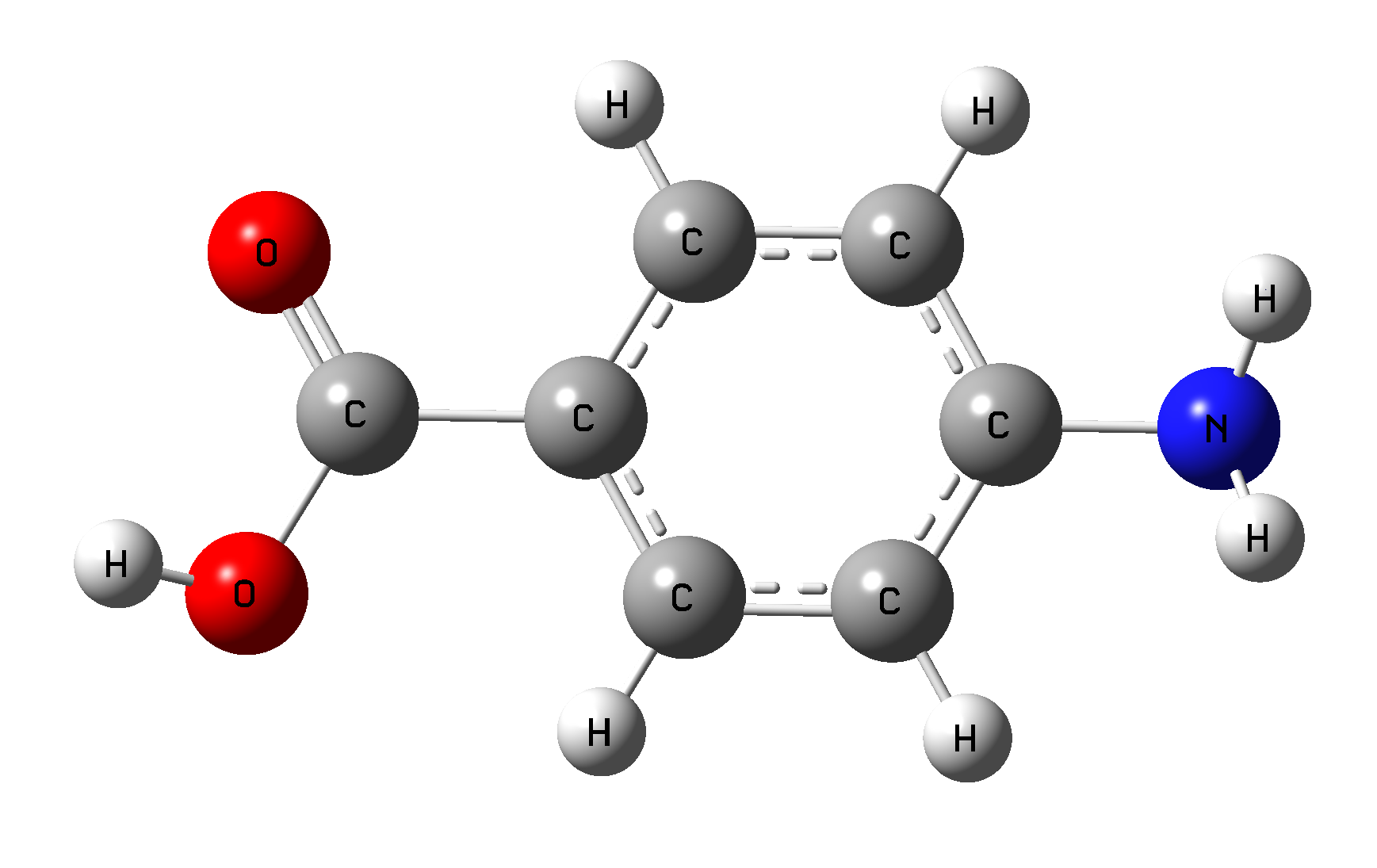

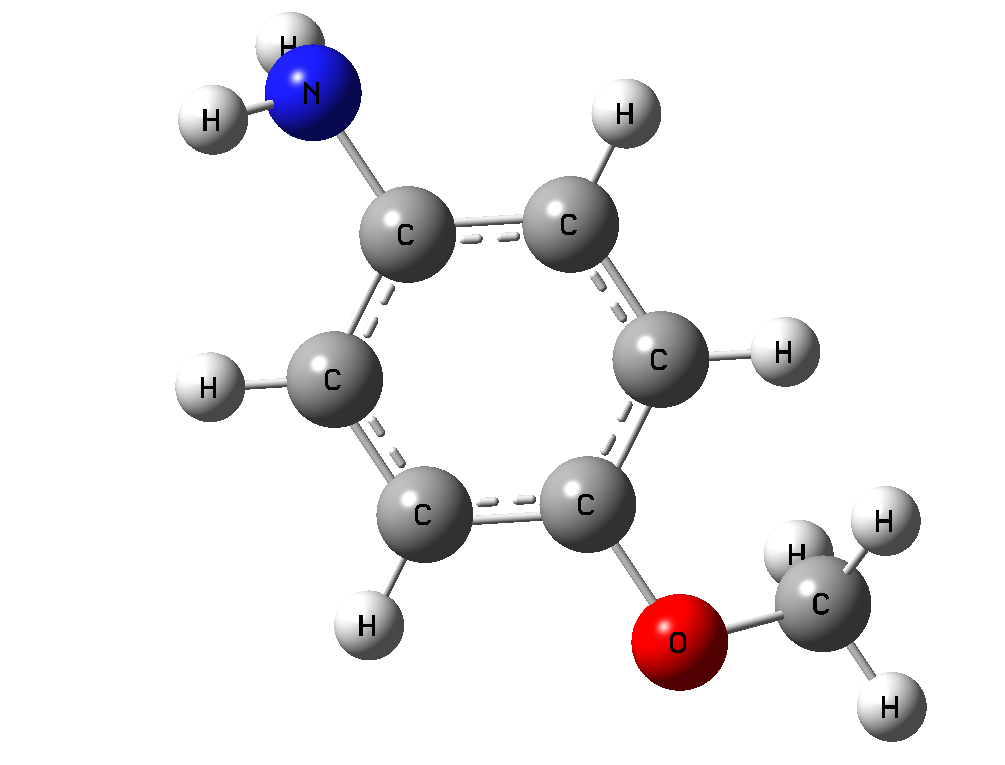

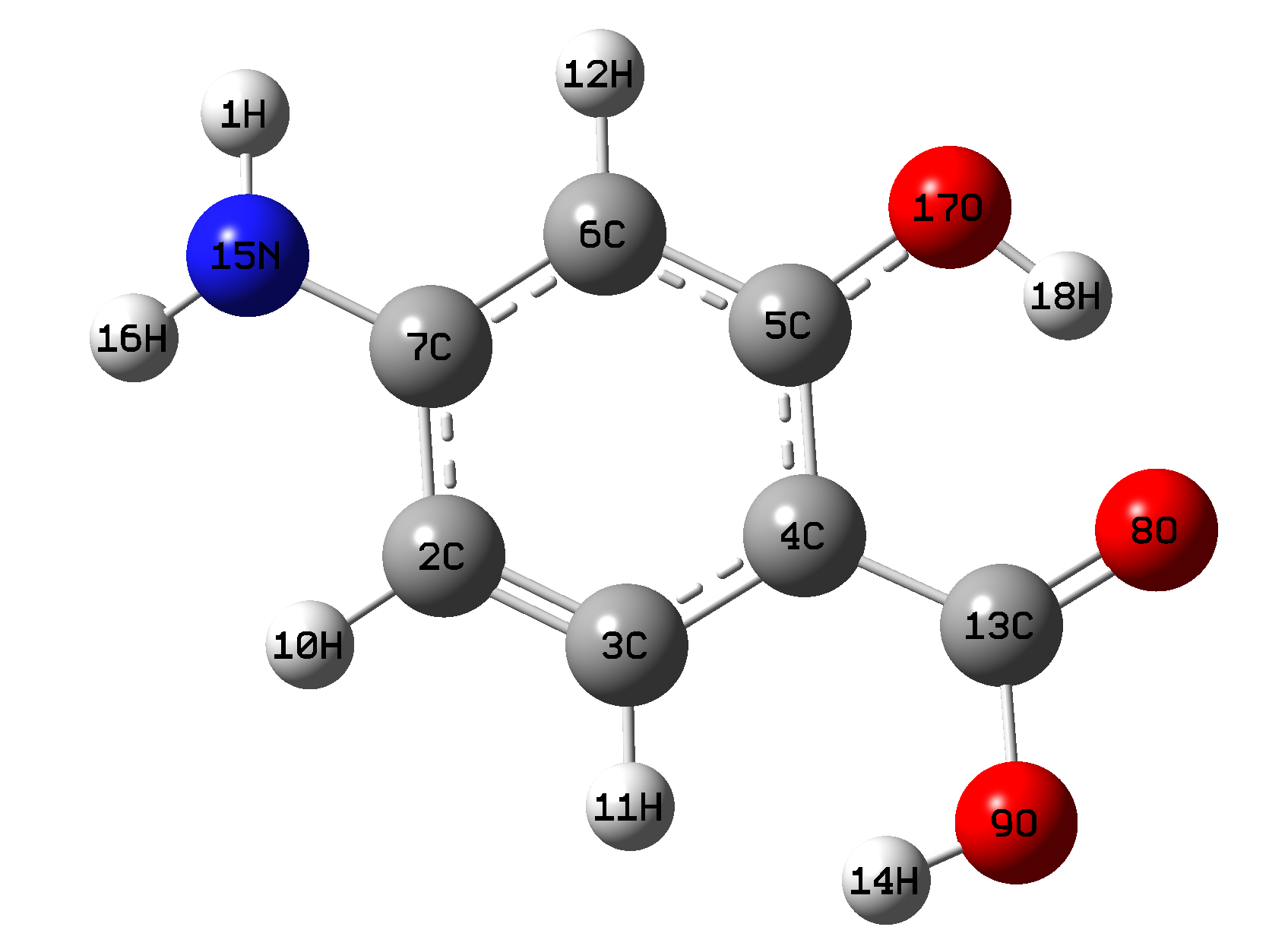


*p-*aminobenzoic acid (PABA) 4-methoxylaniline (4-MA) 4-aminosalicylate (4-AS)


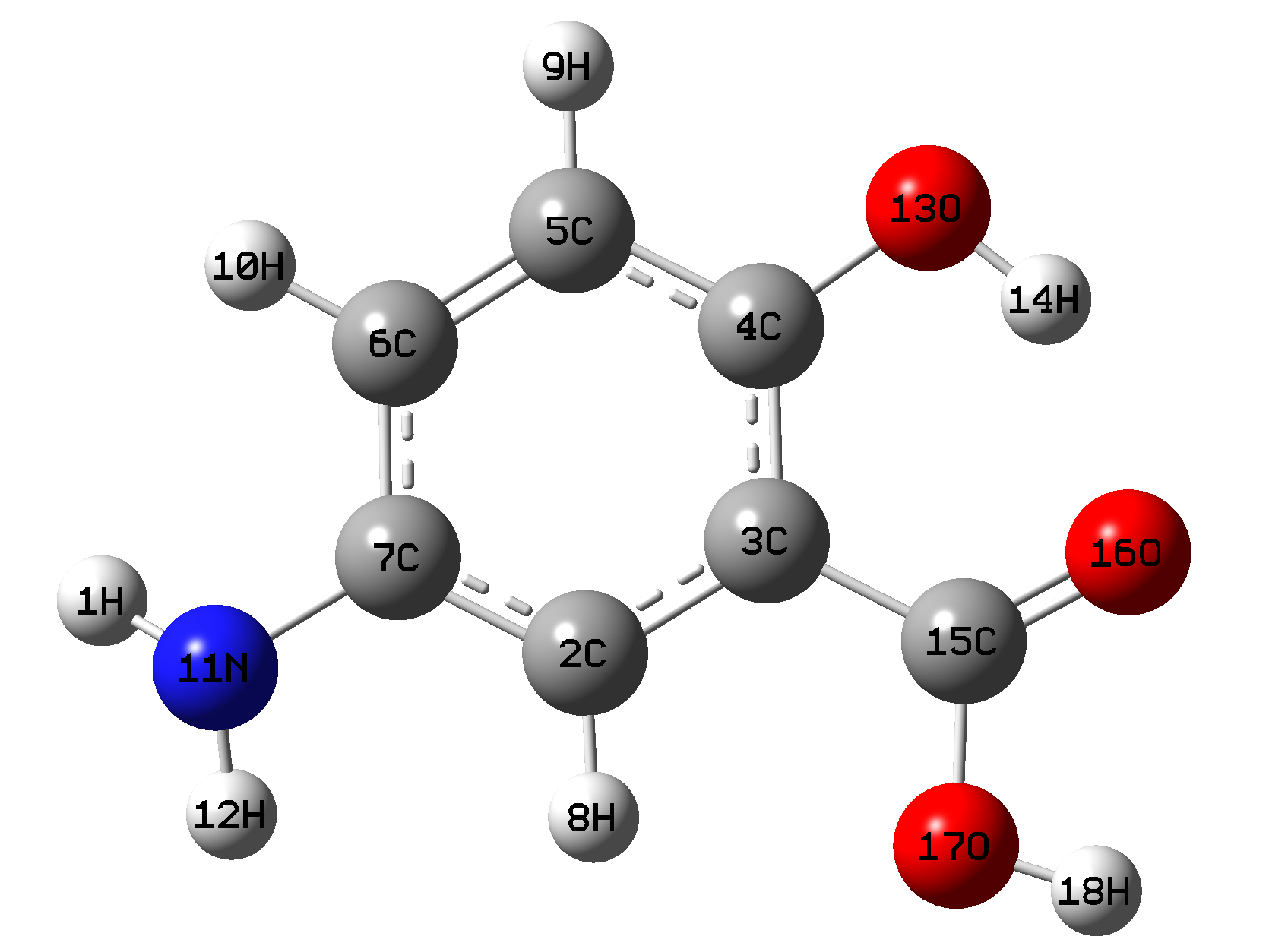

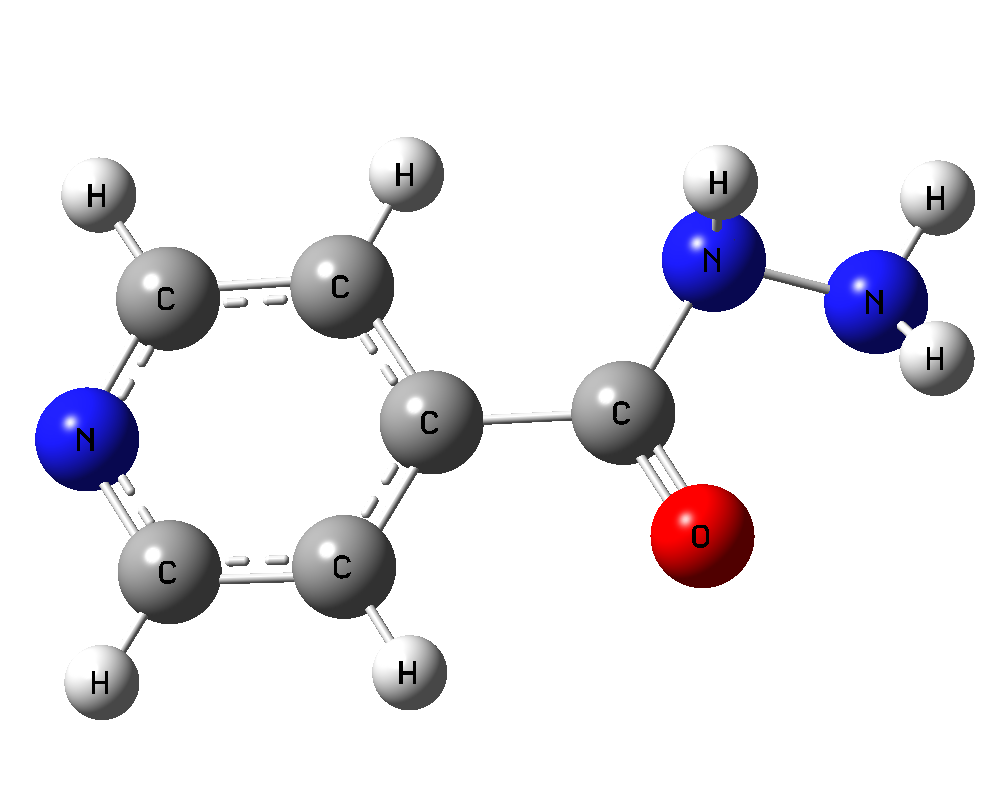

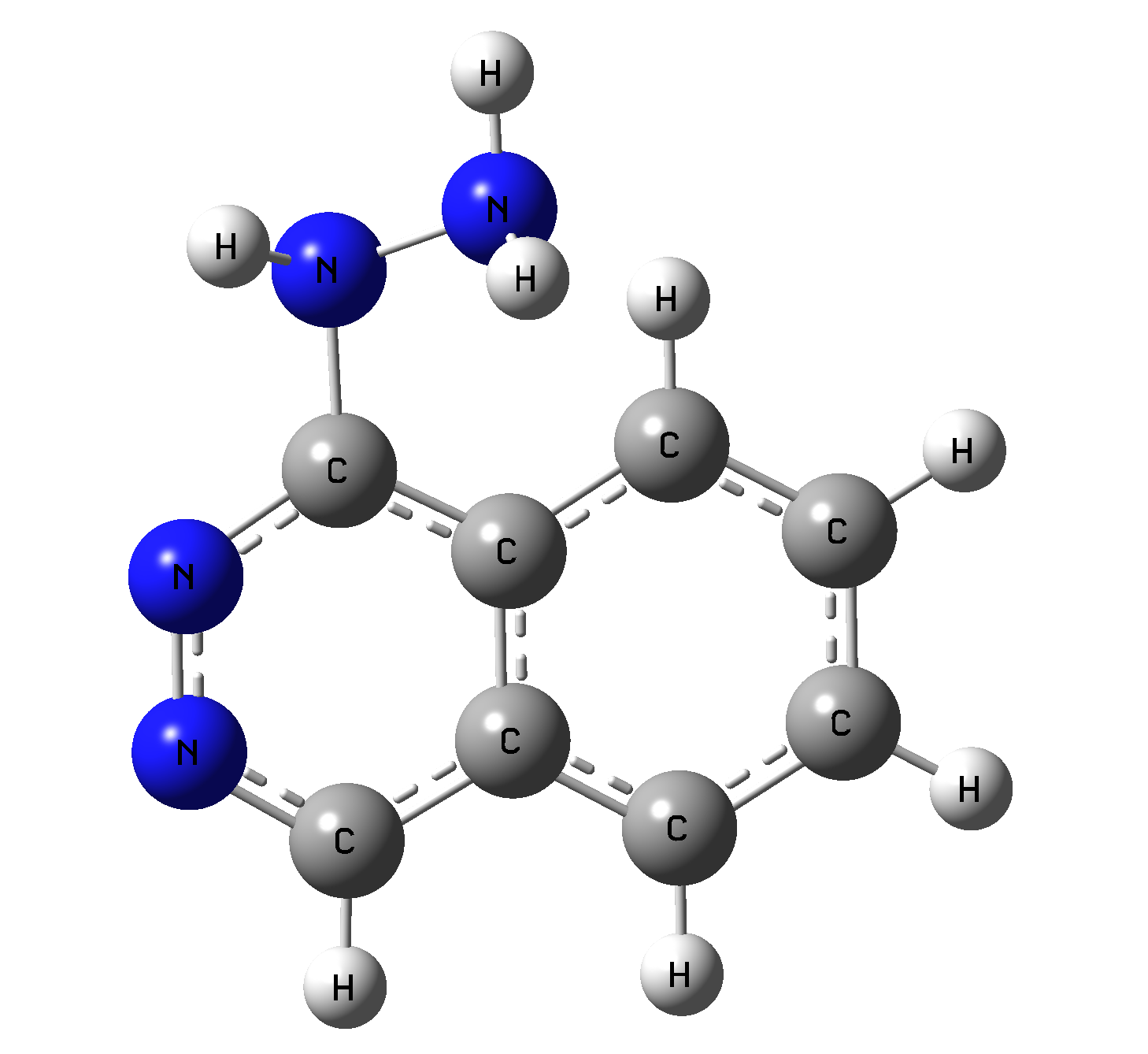


5-aminosalicylate (5-AS) isoniazid(INZ) hydralazine (HDZ)

Fig. 1 The structures of arylamines and arylhydrazines substrates

Table 1 The main structure data for the transition states

| Bond length(Å) | con-ts | | | | stp-ts1 | | | | stp-ts2 | | | |
| --- | --- | --- | --- | --- | --- | --- | --- | --- | --- | --- | --- | --- |
|  | S1C2 | C2N4 | N4H5 | S1H5 | C2O3 | O3H5 | N4H5 | C2N4 | S1C2 | C2O3 | O3H5 | S1H5 |
| PABA | 2.675 | 1.638 | 1.130 | 1.947 | 1.333 | 1.326 | 1.238 | 1.643 | 2.767 | 1.319 | 1.142 | 1.983 |
| 4-MA | 2.756 | 1.615 | 1.100 | 1.980 | 1.342 | 1.323 | 1.253 | 1.600 | 2.786 | 1.317 | 1.029 | 2.023 |
| 4-AS | 2.674 | 1.640 | 1.112 | 1.938 | 1.332 | 1.328 | 1.238 | 1.643 | 2.786 | 1.317 | 1.029 | 2.023 |
| 5-AS | 2.697 | 1.628 | 1.095 | 1.992 | 1.335 | 1.327 | 1.238 | 1.623 | 2.767 | 1.323 | 1.034 | 2.011 |
| INZ | 2.689 | 1.602 | 1.131 | 1.870 | 1.324 | 1.350 | 1.239 | 1.650 | 2.733 | 1.333 | 1.037 | 2.021 |
| HDZ | 2.821 | 1.566 | 1.143 | 1.940 | 1.326 | 1.352 | 1.244 | 1.621 | 2.842 | 1.322 | 1.009 | 2.117 |
| Bond angle(°) | con-ts | | | | stp-ts1 | | | | stp-ts2 | | | |
|  | C2S1H5 | S1C2N4 | C2N4H5 | N4H5S1 | N4C2O3 | C2O3H5 | C2N4H5 | O3H5N4 | H5S1C2 | S1C2O3 | C2O3H5 | O3H5S1 |
| PABA | 48.5 | 79.3 | 92.1 | 137.7 | 94.5 | 78.0 | 69.4 | 117.8 | 41.7 | 77.1 | 102.1 | 131.8 |
| 4-MA | 47.2 | 78.2 | 94.6 | 138.3 | 94.9 | 78.0 | 70.8 | 115.2 | 42.1 | 78.2 | 102.3 | 130.8 |
| 4-AS | 48.5 | 79.0 | 91.8 | 137.4 | 94.5 | 77.9 | 69.3 | 117.7 | 41.6 | 77.6 | 103.4 | 129.5 |
| 5-AS | 48.1 | 79.7 | 93.6 | 136.9 | 94.9 | 77.8 | 70.1 | 117.0 | 41.7 | 78.0 | 102.1 | 131.7 |
| INZ | 48.6 | 78.4 | 93.2 | 139.5 | 92.9 | 79.9 | 71.3 | 113.5 | 42.3 | 79.3 | 101.0 | 130.6 |
| HDZ | 46.4 | 76.1 | 97.9 | 139.6 | 93.7 | 79.4 | 71.9 | 112.6 | 40.7 | 75.6 | 103.1 | 130.2 |

Table 2 The relative energies for different pathways

| Energy/a.u | reactants | con-ts | stw-ts1 | intmed | stw-ts2 | products |
| --- | --- | --- | --- | --- | --- | --- |
| PABA | 0.0 | 176.2 | 203.0 | 49.1 | 130.2 | -17.3 |
| 4-MA | 0.0 | 154.3 | 181.2 | 28.9 | 125.5 | -40.3 |
| 4-AS | 0.0 | 172.1 | 205.9 | 47.0 | 132.9 | -22.0 |
| 5-AS | 0.0 | 142.9 | 184.0 | 42.4 | 114.5 | -18.6 |
| INZ | 0.0 | 141.7 | 182.2 | 26.0 | 110.3 | -39.4 |
| HDZ | 0.0 | 114.8 | 198.3 | 25.7 | 122.1 | -22.9 |
